# Proteomic identification of secreted proteins as surrogate markers for signal transduction inhibitor activity

**DOI:** 10.1038/sj.bjc.6603544

**Published:** 2007-01-09

**Authors:** C M McClelland, W J Gullick

**Affiliations:** 1 Cancer Biology Laboratory, Department of Biosciences, University of Kent, Canterbury, Kent CT2 7NJ, UK

**Keywords:** surrogate marker, signal transduction, epidermal growth factor receptor, proteomics, Gefitinib

## Abstract

Epidermal growth factor receptor is a potential target for cancer treatment and new small-molecule tyrosine kinase inhibitor drugs have been designed to inhibit its activity. In this work we identify potential surrogate markers of drug activity using a proteomic analysis. Two-dimensional electrophoresis was optimised to compare expression patterns of proteins secreted from the cancer cell lines A431 and A549 treated with Gefitinib (Iressa) *vs* untreated or vehicle-only-treated samples. Upregulated or downregulated proteins were detected using Phoretix 2D image analysis software. Several proteins were then identified using matrix-assisted laser desorption/ionization-time of flight (MALDI-TOF) mass spectrometry. In one case, upregulation of Protein Disulphide Isomerase in response to Gefitinib was confirmed by Western blot analysis, and the response was shown to be concentration dependent. The identification of surrogate markers may be of use for the evaluation of new drugs, in preclinical models, in clinical trials and in the therapy of individual patients to give optimal biological drug doses.

Cancer is a collection of different diseases with the common feature of uncontrolled cell growth. Treatment is complicated since the cancer exploits the natural intracellular signalling and metabolic pathways of the cell (Hahn and Weinberg, 2002), often making treatments toxic to both healthy and cancerous cells. The study of the molecular biology of cancer has identified several proteins which have potential as targets for new anticancer drugs (Sebolt-Leopold and English, 2006) and, in some cases, laboratory preclinical experiments have provided support that has encouraged the development of drugs directed against these systems. These new agents produce a more selective effect on these cells as they are directed, in principle, specifically to the molecular changes which distinguish cancer cells from normal cells thereby reducing toxicity to normal tissues (Benson *et al*, 2006). A few drugs to these targets have been evaluated by clinical trials, and a fraction of these have received regulatory approval for use in certain groups of cancer patients (Garber, 2006). Those that have progressed along this path have proved to be quite efficacious and to have low toxicity. It is generally acknowledged, however, that a more efficient process of evaluation and selection would expedite this process making these more useful drugs available at earlier times and potentially at lower cost.

Much discussion has taken place about the potential value of surrogate markers for drug activity (Ross *et al*, 2005). Surrogate markers are proteins or other molecules expressed in response to a disease state or a drug, to predict a true end point, which in cancer is patient survival. They also have potential value in preclinical work allowing sequential measurements to be made in animal models and in estimation of the optimal biological dose in mouse and man. They may also be of use in similar studies in phase I clinical trials and in the longer term to optimise drug dose for individual patients. Despite the desire to obtain such reagents, the literature does not contain many examples of such reports.

A particular class of targets, which have proved to be of value are the proteins involved in signal transduction (Benson *et al*, 2006). The family of signal transduction proteins that has received the most attention to date are the Epidermal Growth Factor Receptor (EGFR) family, most likely as several of its members are manifestly involved in stimulating tumour growth and concepts of drug action are clear (Yarden and Sliwkowski, 2001; Mosesson and Yarden, 2004; Warren and Landgraf, 2006). Examples of drugs to these include antibodies to growth factors receptors such as Cetuximab and Trastuzumab and small molecule tyrosine kinase inhibitors such as Gefitinib (Iressa), Erlotinib (Tarceva) and Lapatinib (Tykerb) (Normanno *et al*, 2004; Baselga and Arteaga, 2005).

Some research has been undertaken to develop surrogate markers for these agents. One landmark study describes a pharmacodynamic measurement of the phosphorylation state of the EGFR in normal skin taken by biopsy from patients involved in phase II trials of the drug Gefitinib, as a surrogate of the activation state of the protein and therein the activity of the drug (Albanell *et al*, 2002). Although this was one of the first studies of this kind, it has the practical disadvantage that serial measurements are difficult to obtain for ethical reasons. A more generally useful measurement would be a serum test as a surrogate for drug activity (Baselga, 2003). One example of this approach has been the measurement of the levels of pro-angiogenic factors in the serum from patients treated with an inhibitor of both VEGF and PDGF receptors, sunitinib malate, where levels of VEGF-A and PIGF increased and sVEGF-R2 levels decreased (Motzer *et al*, 2006).

Growth factor receptor signalling is known to regulate the expression of many genes, and some of these encode secreted proteins. We hypothesise that inhibiting receptor signalling will alter this balance and that it should be possible to detect such changes and then develop them as an indirect quantitative assay for drug action. In this paper, we present work using a proteomic method to discover proteins secreted from model cancer cell lines whose expression is modulated (up or down) by the signal transduction inhibitor Gefitinib. These were detected using two-dimensional gel electrophoresis and identified by mass spectrometry. In one case, further experiments using antibodies to detect expression in complex protein mixtures showed that this altered expression was drug dose dependent. Clearly these initial results need to be extended to look for proteins with this behaviour which are shared by most or all cancers of a particular type and which can be monitored in a sensitive serum based assay. Such a test can then be applied to serum obtained from patients during drug trials to see if the assay has general utility.

## MATERIALS AND METHODS

### Cell culture

A431 and A549 cells were obtained from Cancer Research UK (London, UK) and grown as adherent monolayers in Dulbecco's modified Eagle's medium (DMEM) supplemented with 10% Foetal Calf Serum (FCS), 1% L-Glutamine and 1% penicillin–streptomycin (Invitrogen, Paisley, Scotland).

### Preparation of concentrated conditioned media to run on 2D gels

The following optimised method for preparation of concentrated conditioned media was used. Thirty dishes (10 cm diameter) of A431 or A549 cells were grown to 70% confluency. A series of washes were performed according to Sato *et al*, (2003), consisting of three washes with serum-free medium (0%), 1 h incubation at 37°C in serum-free medium and then two more washes with serum-free medium,and were refreshed with 2 ml of fresh serum-free medium. Ten dishes of cells (∼8.8 × 10^7^ cells each) were treated with 5 × 10^−6^ M Gefitinib, with an equal volume of DMSO (vehicle), or were left untreated. All dishes were then incubated at 37°C. Conditioned media were collected after 72 h, pipetted into 1.5 ml Eppendorfs and centrifuged for 10 min at 16 000 **g** to remove cell debris. Samples were then concentrated using Vivascience concentrators with polyethersulfone (PES) membranes and a filter cutoff point of 10 000 MW using a Sanyo centrifuge for 40 min at the speed recommended for the concentrator, then the concentrators were reverse -centrifuged into the concentrate recovery cap for 4 min to collect the sample. Protein concentrations were determined by assaying samples using the standard Bradford assay (Bradford, 1976). The samples were then diluted with an isoelectric focusing buffer (ultra pure urea, 9.5 M in ddH_2_O (Milli-Q water), 2% w v^−1^ of CHAPS, 1% w v^−1^ of DTT and 0.8% of pharmalytes (pH 3–10), 1% v v^−1^ phosphatase inhibitor cocktail 2 and 4.9 mg ml^−1^ of protease inhibitor cocktail).

### Running the first dimension – isoelectric focusing

The first dimension was run using an IPGphor from Amersham Biosciences (Little Chalfont, UK) using 18 cm pH 3–10 (NL) immobilised pH gradient (IPG) strips. A 1 mg volume of protein in 350 *μ*l of isoelectric focusing buffer was run under the following conditions: instrument temperature 20°C; maximum 50 *μ*A strip; rehydration, step 1, 30 V for 12 h; step 2, step-n-hold at 200 V for 1 h; step 3, step-n-hold at 500 V for 1 h; step 4, gradient at 8000 V for 1 h; step 5, step-n-hold at 8000 V for 4 h; step 6, step-n-hold 8000 V until the total volt hours reached 32 000. After the run was completed, the strips were washed in ddH_2_O (Milli-Q water) and stored in 10 cm tissue culture pipettes at −70°C until the second dimension was run.

### Second Dimension – SDS–PAGE

SDS–PAGE gels (12%) were utilised for the second dimension. Strips were equilibrated and then placed on the second dimension gel and run using the following settings: a constant voltage of 250 V and settings of 300 W and 50 mA, until just before the dye front reached the bottom of the gel. After completion of the run, the gels were stained with Coomassie® Blue G250 stain from Bio-Rad (Hemel Hempstead, UK). Gels were then destained with ddH_2_O for 2 h. A digital image of gels was captured using a calibrated scanner, ImageScanner™, a flatbed optical scanner from Amersham Biosciences using LabScan software from Amersham Biosciences at 600 dpi and 256 greyscales, and true colour in transmissive mode. The scanner was calibrated using a Calibration OD step tablet from Genomic solutions Ltd. (Huntingdon, UK).

### Image analysis

The twelve 2D gels of A431 and the 12 gels of the A549 concentrated conditioned media were analysed using Phoretix™ 2D software version 2003.02, Non-Linear Dynamics (Newcastle upon Tyne, UK) (Mahon and Dupree, 2001). Average gels were created from the four gels for each type of treatment: ‘Not treated’, ‘DMSO (drug vehicle) treated’ and ‘Gefitinib’ treated, and the maximum number of gels where the spots may be absent were one in four. Normalisation was then undertaken and then statistical analysis of the results was performed to display the standard deviation of the normalised spot volumes, and a Student's *t*-test was undertaken to compare Gefitinib-treated and the DMSO control spots.

### Matrix-assisted laser desorption/ionization-time of flight(MALDI-TOF) mass spectometry

#### Preparation of protein spots from 2D gels for MALDI-TOF analysis

This method was an adaptation of the original Shevchenko method (Shevchenko *et al*, 1996). Briefly, a 1 ml pipette tip trimmed to the size of the spot was used to cut out colloidial Coomassie-stained spots from 2D SDS–PAGE gels. Spots were placed in 0.5 ml Eppendorf tubes prewashed with methanol and destained using 100 mM ammonium bicarbonate in 50% acetonitrile. Samples were then washed three times in 50% acetonitrile in ddH_2_O (Milli-Q water) and dried in a speed vac from ThermoSavant (Holbrook, USA). The reduction of disulphide bonds was performed by adding 10 mM DTT (in 5 mM ammonium bicarbonate, pH 8.0) and then incubating the samples for 45 min at 50°C in a heat block. Alkylation of cysteines (Herbert *et al*, 2001) was performed by adding 50 mM Iodoacetamide (in 5 mM ammonium bicarbonate, pH 8.0) and then incubating the samples for 1 h at room temperature in the dark. The gel pieces were washed twice in 50% acetonitrile and then dried. To perform the in-gel digestion, ∼7 *μ*l of sequencing grade-modified porcine trypsin was added to each gel piece at RT at a concentration of 20 *μ*g ml^−1^ in 5 mM ammonium bicarbonate (pH 8.0). After 5 min gel pieces were overlaid with 5 *μ*l of 5 mM ammonium bicarbonate (pH 8.0) and incubated overnight at 37°C. The peptides were extracted by centrifuging the tubes to spin liquid down from the lid. In total,10 *μ*l 50% acetonitrile/5% trifluoroacetic acid (TFA) in ddH_2_O (Milli-Q water) was added to the supernatant and it was then transferred to fresh Eppendorf tubes. This extraction was repeated twice and after each extraction, the supernatant was added to the new Eppendorf tubes. The samples were then concentrated by drying in a speed vac. The samples were stored at −20°C until mass spectrometry was performed.

#### Analysis of peptides using MALDI-TOF mass spectrometry

Peptides were resuspended in 5 *μ*l of 0.1% formic acid in ddH_2_O (Milli-Q water). The matrix used was saturated using 2,5-dihydroxybenzoic acid (DHB) solution in 0.5 ml in ddH_2_O (Milli-Q water). Onto the MALDI target 0.75 *μ*l portion of the matrix was spotted, and 0.75 *μ*l of sample was spotted on top, before the matrix dried. MALDI-TOF mass spectra were obtained using an Ultraflex MALDI-TOF MS from Brucker Daltonik (Bremen, Germany) in reflectron mode with external calibration using peptide mix standards from Brucker Daltonik (Bremen, Germany). Peptide masses were submitted to an analysis programme, Flex analysis from Brucker Daltonik (Bremen, Germany). Peptide masses from the trypsin digestion were entered into the Mascot search engine (Perkins *et al*, 1999) to identify proteins. Within Mascot, four different databases were available to search from, NCBInr, MSDB, SwissProt and OWL. Proteins were first analysed at 50 p.p.m. and then at lower sensitivities if an identification was not made. Proteins that did not obtain significant protein identification were not analysed further.

#### Confirmation of protein disulphide isomerase (PDI) upregulation in conditioned medium of A431 cells in response to Gefitinib using Western blotting

Five 10-cm diameter plates of A431 cells were grown to 70% confluency. Serum-free media washes were carried out as before. Cells were then treated with Gefitinib at 5 × 10^−6^ M, 1 × 10^−5^ M, a DMSO only control, for each concentration of Gefitinib or cells were not treated. Conditioned medium was collected and concentrated as above. The standard Bradford assay was performed to determine the total protein concentration, 2 × sample buffer was added to the samples to obtain an equal protein load on 9% SDS–PAGE gels. Proteins were transferred onto PVDF membranes overnight. Blots were shaken in blocking buffer consisting of 5% w v^−1^ Marvel (nonfat milk powder) from Premier Brands (Spalding, UK) in PBS/0.1% v v^−1^ polyoxyethylenesorbitan monolaurate (Tween 20) for 1 h and washed in PBS/0.1% Tween 20 (5 × 5 min washes). Blots were then probed for PDI using a rabbit anti-PDI polyclonal antibody SPA-890 from Stressgen (Victoria, Canada) diluted to 1:1000 of the original stock in PBS/0.1% Tween 20 for 1.5–2 h. Blots were then washed in PBS/0.1% Tween 20 (5 × 5 min washes), probed with the secondary antibody, which was a swine anti-rabbit antibody conjugated to horse radish peroxidase (HRP) from DAKO (Cambridgeshire, UK) at a final concentration of 1.3 *μ*g ml^−1^ made up in PBS/0.1% Tween 20. After 1 h, five final washes were undertaken in PBS/0.1% Tween 20 (5 × 5 min washes), and the signal was developed using enhanced chemiluminescent (ECL) plus from Amersham Biosciences (Little Chalfont, UK) using a compact x4 hyperprocessor from Xograph imaging systems (Tetbury, UK). The experiment was repeated to show that the results were reproducible.

## RESULTS

Two cell lines were chosen for this work, A431 cells derived from a vulval squamous cell cancer and A549 cells from an adenocarcinoma of the lung. These were selected as they are known to express high levels of the EGFR (A431 2 × 10^6^ and A549 5 × 10^5^ respectively) and to be dependant on activity of the EGFR for sustained growth. Moreover, A431 cells are a well established model in EGFR research, and the A549 cells are from a common cancer type in which the effects of Gefitinib have been evaluated in clinical trials. We first tested their sensitivity to Gefitinib by treating A431 cells with EGF in the absence or the presence of increasing drug concentrations. Gefitinib is rather insoluble in water, so this was dissolved in DMSO and, thus, a vehicle-only control was included in all subsequent experiments. There was, as predicted, a progressive inhibition of receptor phosphorylation on tyrosine with increasing drug dose as previously reported. Substantial inhibition was seen at 5 × 10^−6^ M Gefitinib, which was therefore used in subsequent experiments.

**Figure 1:**
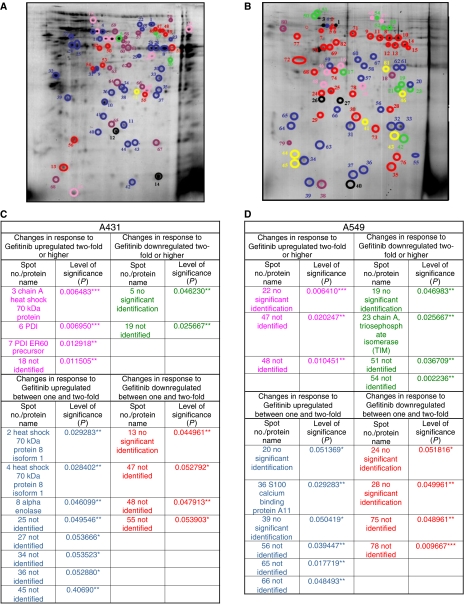
Results of image analysis undertaken of an average (**A**) A431 and (**B**) A549 Colloidal Coomassie Blue-stained 2D gel of concentrated conditioned medium displaying all the protein spots which are upregulated 2-fold (pink), upregulated 1-2-fold (blue), downregulated two-fold (green) or downregulated between 1- and 2-fold (red) in response to Gefitinib. Spots missing from Gefitinib-treated gels are marked in yellow and spots missing from not-treated gels and DMSO-treated gels (purple). A few example spots which do not change in response to Gefitinib are also marked using black circles. Protein spots from (**C**) A431 and (**D**) A549 cells which display significant changes in protein expression in response to Gefitinib compared with the DMSO vehicle control using the Student's *t*-test at the ^***^P<0.01, ^**^P<0.05 or ^*^P<0.1 level and which have been analysed using MALDI-TOF mass spectrometry.

Cells were grown and treated with DMSO alone, Gefitinib was dissolved in DMSO or was untreated for 72 h and serum-free conditioned medium containing secreted proteins was prepared for 2D gel separation, as described in the Materials and Methods section. Isoelectric focusing was undertaken using 18 cm immobilised nonlinear pH 3–10 gradient strips, and the second dimension separation was performed using 12% denaturing SDS gels. In each case, four replicate gels were run for each condition. The separated proteins were detected using colloidal coomassie blue G250 staining, and digital images of the gels were acquired. Figure 1A and B show gels of proteins secreted from the A431 and A549 lines. Reference gels were created for each condition, and the Phoretix software was used to detect spots which were upregulated or downregulated significantly between cells treated with vehicle only or the drug in DMSO, and these were divided into those whose expression varied up to 1–2-fold and those which changed two-fold or more. The proteins selected using the Phoretix software were analysed further using the Student's *t*-test, and the protein spots shown to display significant changes using both types of analysis were prepared for mass spectrometry (Figure 1C and D). From the Student's *t*-test analysis, six protein spots were identified from conditioned medium from A431 cells with a significant change in expression of more than two fold, four were upregulated and two were downregulated. Twelve spots which varied by 1–2-fold, were detected eight were reduced and four increased. Seven protein spots from conditioned medium from A549 cells were altered more than two-fold (three upregulated and four downregulated) and ten spots changed 1–2-fold (six upregulated and four downregulated).

We next sought to identify each of the proteins which were changed in expression significantly according to the Student's *t*-test. Individual protein spots were excised from the gels and subject to tryptic digestion and MALDI-TOF mass spectrometric analysis. Mascot software was employed to identify proteins from a comparison of their mass fingerprints with up to four databases as described in the Materials and Methods section and those with a significant score are listed (Figure 1C and D and Supplementary Tables 1 and 2).

**Figure 2:**
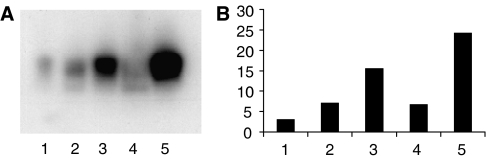
(**A**) Western blot to determine PDI levels in conditioned media of A431 cells treated with, track 1, no treatment; track 2, DMSO control; track 3, 5 × 10^−6^ M Gefitinib; track 4, DMSO control; track 5, 1 × 10^−5^ M. (**B**) Image analysis of the density of the autoradiographic signal expressed in arbitrary units: 1, no treatment; 2, DMSO control; 3, 5 × 10^−6^ M Gefitinib; 4, DMSO control; 5, 1 × 10^−5^ M.

One protein from A431 cells gave a particularly large increase in expression levels. This was identified by MALDI-TOF mass spectrometry (at a significant score) as protein disulphide isomerase. In order to provide independent validation of the original experiments, we treated new cultures of cells and again collected conditioned medium using the two drug concentrations, 5 × 10^−6^ M and 1 × 10^−5^ M. Samples of untreated, vehicle–only-treated and drug in DMSO-treated cell conditioned medium, were analysed using Western blotting, and an antibody specific for human PDI was utilised to detect the expression of this protein in the mixture. Protein disulphide isomerase was upregulated as seen in the 2D gels, and this was proportional to the concentration of the drug. Scanning the autoradiograph to estimate the effects on expression levels showed an approximately six-fold induction of expression at the highest level of drug added (Figure 2).

## DISCUSSION

In this work using a model system, we have shown that it is possible to detect reproducible changes in expression levels of a range of proteins secreted from cancer cells. Two processes of statistical validation of the identification of individual proteins were applied. First, the change in level of expression of equivalent spots had to reach significance using the standard deviation of the normalised spot volumes and also a Student's *t*-test comparing Gefitinib-treated and DMSO-treated gels. Second, the identification of the proteins from this set of spots as known proteins using MALDI-TOF mass spectrometry and the Mascot search engine had to be significant at 50 p.p.m. (the detailed data are presented in Supplementary Tables 1 and 2). After this rigorous selection process, six of the 12 proteins upregulated by Gefitinib in A431 cells were identified. Three spots were identified as members of the heat-shock family HSP70 protein family. Spots 2 and 4 were both heat shock protein 70 protein 8 isoform 1, and the other, spot 3, was chain A of heat shock 70. Heat-shock proteins (HSP) are comprised of four major families, the small HSP (sHSP) family, the HSP60 family, the HSP70 family and the HSP90 family, and these families are classified according to the molecular weight of the protein. Therefore, heat shock 70-kDa protein 8 isoform 1 belongs to the HSP70 family. The HSP70 family is induced by stress, for example, caused by the effect of a drug or disease, and HSP70 is upregulated in many cancers (Lichtenfels *et al*, 2002) including breast, gastric, prostate and colorectal cancer. Heat-shock protein 70 is also involved in correct protein folding, assembly of newly synthesised proteins and the disassembly of protein aggregates. Heat-shock proteins are also regulators of apoptosis which could be occurring in response to Gefitinib. Heat-shock proteins were originally reported to be located in the cytoplasm or ER of the cell, but HSP70 is found on the cell surface (Shin *et al*, 2003) and in normal peripheral blood (Wehner *et al*, 2003). The heat-shock 70-kDa protein 1 (HSP70.1) is also a member of the HSP70 family and has a number of roles in the cell including, chaperon function, stabilising proteins against aggregation and facilitating protein folding in the cytosol and in organelles (Milner and Campbell, 1990). Heat-shock 70-kDa protein 1 (HSP70.1) and three other members of the HSP70 family (GRP78, HSC70 and GRP75) are upregulated in cancerous tissue (Takashima *et al*, 2003). Consistent with our data Shin *et al*, (2003) have studied the global cell surface proteome of a number of cancer cells (including the A549 cell line) and found a surprising abundance of chaperones including GRP78, GRP75 and notably HSP70, HSP60, HSP54 and HSP27.

Two further proteins upregulated in A431 conditioned medium are PDI and its precursor PDI ER60. Protein disulphide isomerase is involved within the ER in folding proteins which contain disulphide bonds (Freedman *et al*, 1994; Jiang *et al*, 1999; Herbert *et al*, 2001). In addition, it has been shown that PDI can protect neurons and endothelial cells from hypoxic cell death (Sullivan *et al*, 2003). It is also located in other organelles and is also secreted from the cell (Yoshimori *et al*, 1990). ER60 (Erp60), also called Erp57, is a protein disulphide isomerase precursor, a human glucose-regulated protein and the closest-known homologue of PDI (Frickel *et al*, 2004) sharing 33% of the same amino acids as PDI. ER60 is expressed in response to conditions of stress (Ferrari and Soling, 1999). ER60 was found to show a two-fold upregulation in response to Gefitinib compared with untreated and DMSO-treated A431 cells conditioned medium.

Finally, in conditioned medium from A431 cells, the protein alpha enolase was found to be induced 1–2-fold. Alpha enolase is a cytoplasmic glycolytic enzyme (Chang *et al*, 2003) and is downregulated in non-small-cell lung carcinoma, and so the upregulation of this protein with Gefitinib could play a part in the reversal of tumorigenesis. Alpha enolase belongs to a group of cell-surface proteins (Pancholi, 2001) and has been proposed as a biochemical marker in myocardial damage detection (Mair, 1997) and a neuron-specific form of the protein has been proposed as a tumour marker in oncology (Jacobs and Haskell, 1991).

Two proteins were identified in the secretome of A549 cells, the A chain of triosephosphate isomerase (TIM) (downregulated >2-fold) and the S100C calcium-binding protein A1 (upregulated 1–2-fold). The upregulation of S100C in response to Gefitinib was confirmed by Western blotting (data not shown). Triosephosphate isomerase (TIM) is an enzyme which catalyses the interconversion between D-glyceraldehyde-3-phosphatate and dihydroxyacetone phosphate. An autoantibody to TIM is found in the serum of patients with osteoarthritis (Xiang *et al*, 2004) and in patients with neuropsychiatric lupus (Watanabe *et al*, 2004). Triosephosphate isomerase was also found to be significantly overexpressed in lung adenocarcinoma consistent with its detection in the conditioned medium of the A549 cell line (Chen *et al*, 2002). S100C is a calcium-binding protein involved in cell cycle progression and differentiation, which is localised in the cytoplasm and nucleus of many cell types. This protein has been shown to be downregulated in a range of malignant tissues, compared to normal tissues including the bronchus, mammary duct, renal tube, prostate, uterus and testis. therefore, this protein could be a candidate for a new tumour marker (Kondo *et al*, 2002). S100C secretion is upregulated in response to Gefitinib and, therefore, Gefitinib could be, in part, reversing the malignant phenotype.

Collectively, the proteins identified here are involved in the cells response to stress. Although Gefitinib was designed to target specifically the EGFR, it is now clear that it can bind to and inhibit with differing affinities, at least 27 kinases (Brehmer *et al*, 2005). It is likely that when other SMTKIs are examined in more detail they will also have a range of ‘on-target’ and ‘off-target’ effects. It will be interesting to see, however, if the responses to them share similarities such as inducing the expression of stress response proteins. If so, these might be useful markers for drug activity since ultimately the desired effect is death of the cancer cells.

In summary, the work presented here demonstrates that it is possible to detect proteins whose secretion into the medium is altered by treatment with an inhibitor of the EGF receptor. In one case, this appeared to be concentration dependent. Clearly in order to be of practical use, such markers should be secreted by particular cancer types. It may then be possible to develop this approach to make sensitive assays which could be tested on serum obtained from animals bearing xenografted human tumours and in, in the future, patient's serum as a surrogate assay for drug activity.
