# Supplementary material for: Proteomic identification of secreted proteins as surrogate markers for signal transduction inhibitor activity
Source: Br J Cancer. 2007 Jan 9;96(2):284–9. doi: 10.1038/sj.bjc.6603544 (PMC2360009; doi:10.1038/sj.bjc.6603544)
Supplement: Supplementary Tables 1 and 2 [file 6603544x1.doc]

**Supplementary Tables**

Proteins identified with significant protein scores (P<0.05) from Mascot searches of peptide mass fingerprints to identify proteins from 2D SDS PAGE gels.

**Supplementary Table 1.** A431 concentrated conditioned media Mascot searches using the NCBInr database and or Swiss Prot, Owl and MSDB databases.

| Supplementary Table 1 Significant protein scores (P<0.05) of Mascot searches of peptide mass fingerprints to identify proteins from 2D SDS PAGE gels of A431 concentrated conditioned media Taxonomy=human Max missed cleavages=0 | | | | | | | | |
| --- | --- | --- | --- | --- | --- | --- | --- | --- |
| **Sample idenfication**  **(Spot no.)** | **Probability based Mowse score** | **Top score protein** | **PI** | **Mr** | **Database searched** | **Accession number**  **of protein** | **Peptide mass tolerance**  **(kDa) or ppm if stated** | **Sequence coverage**  **For sig proteins**  **(%)** |
| Spot 2 | 78 | Heat shock 70 kDa protein 1  (HSP70-1) | 5.48 | 70294 | NCBInr | Gi 462325 | 1 | 21 |
| Spot 2 | 70 | (HS71_  HUMAN)  Splice isoform displayed  (conflict)  Heat shock protein | 5.42 | 70110 | swissprot | PO8107-00-00-01 | 0.65 | 17 |
| Spot 3 | 59 | Heat shock cognate 71 KD protein | 5.37 | 71082 | owl | HS7C_human | 1 | 12 |
| Spot 3 | 69 | Heat shock 70 kDa protein 8 isoform 1 | 5.37 | 71082 | NCBInr | Gi 5729877 | 0.7 | 12 |
| Spot 4 | 64 | Heat shock 70KDa protein 8 isoform 1 | 5.37 | 71082 | NCBInr | Gi 5729877 | 0.5 | 20 |
| Spot 4 | 64 | HS7C_human  Conflict  Heat shock cognate 71KDa protein | 5.37 | 71082 | Swiss prot | P11142-00-00-00 | 0.5 | 20 |
| Spot 6 | 97 | PDI | 6.10 | 57043 | MSDB | S55507 | 1 | 19 |
| Spot 6 | 72 | PDI | 6.23 | 57115 | NCBInr | Gi 2135267 | 400 ppm | 19 |
| Spot 6 | 72 | PDI | 6.23 | 57065 | SwissProt | P30101-00-00-01 | 400 ppm | 19 |
| Spot 7 | 90 | protein disulfide-isomerase (EC 5.3.4.1) ER60 precursor - human | 5.98 | 57160 | NCBInr | gi|7437388 | 1 | 18 |
| spot 8 | 89 | Alpha enolase | 6.99 | 47356 | OWL | ENOA_HUMAN | 2 | 31 |
| Spot 8 | 89 | Alpha enolase | 6.99 | 47356 | MSDB | ENOA_HUMAN | 2 | 31 |
| Spot 8 | 65 | Alpha enolase | 6.99 | 47350 | MSDB | ENOA_HUMAN | 1.5 | 25 |
| Spot 8 | 68 | Alpha enolase | 6.99 | 47350 | Swiss prot | PO6733-00-00-00 | 1.5 | 27 |
| Spot 10 | 73 | Chain A, Glutathione Transferase Human Class Pi Isoenzyme | 5.09 | 23430 | NCBInr | gi|20664358 | 0.9 | 55 |
| Spot 14 | 66 | KIAA0336 | 5.06 | 191474 | NCBInr | Gi 40788217 | 0.6 | 19 |

**Supplementary Table 2.** A549 concentrated conditioned media Mascot searches using the NCBInr database and or Swiss Prot, Owl and MSDB databases.

| **Supplementary Table 2 Significant protein scores (P<0.05) of Mascot searches of peptide mass fingerprints to identify proteins from 2D SDS PAGE gels of A549 concentrated conditioned media Max missed cleavages=0 Taxonomy=human** | | | | | | | | |
| --- | --- | --- | --- | --- | --- | --- | --- | --- |
| **Sample idenfication** | **Probability based Mowse score** | **Top score protein** | **PI** | Mr | **Database searched** | **Accession number**  **of protein** | **Peptide mass tolerance**  **(kDa)** | **Sequence coverage of sig. Proteins (%)** |
| Spot 23 | 69 | Chain A, Triosephosphate Isomerase (Tim) (E.C.5.3.1.1) Complexed With 2-Phosphoglycolic Acid | 6.51 | 26807 | MSDB | TPIS_HUMAN | 0.7 | 45 |
| Spot 23 | 69 | Triosephosphate isomerase (EC 5.3.1.1) (TIM).- Homo sapiens (Human), and Pan troglodytes (Chimpanzee) | 6.51 | 26807 | MSDB | TPIS_HUMAN | 0.9 | 45 |
| Spot 36 | 80 | S100 calcium binding protein A11 (calgizzarin); calgizzarin; S100 calcium-binding protein A11 (calgizzarin); S100 calcium-binding protein A11 [Homo sapiens] | 6.56 | 11847 | NCBInr | gi|5032057 | 0.8 | 42 |
